# Video Intervention to Increase Knowledge About and Interest in Palliative Care Among MSW Students

**DOI:** 10.1093/geroni/igaa057.229

**Published:** 2020-12-16

**Authors:** Cathy Berkman

**Affiliations:** Fordham University, New York, New York, United States

## Abstract

As the population ages and more people live longer with chronic and life-limiting illnesses, more healthcare professionals with palliative care skills are needed. Social workers are part of the palliative care team, but there is little, if any, content on palliative and end-of-life care in MSW programs. A 24-minute video featuring nine palliative and hospice social workers was produced with two goals: 1) increase knowledge of social work students about palliative and end-of-life care; and 2) interest social work students in a career in palliative social work. MSW students from three schools, in NY and Alabama, viewed the video. After viewing the video, 94 students participated in the mixed methods study, completing the brief, anonymous, online survey. The mean level of understanding about what palliative social workers do, rated from 1 (no understanding) to 5 (very good understanding), was 2.96 (SD=.99) before viewing the video and 4.31 (SD=.61) after, for an increase of 1.35 points (95% CI=1.14, 1.55) (p<.001). The mean level of interest in a career in palliative care social work and working with seriously ill persons and their family members, rated from 1 (Not at all interested) to 5 (Extremely interested), was 2.52 (SD=.99) before viewing the video and 3.45 SD=.80) after, for an increase of .91 points (95% CI=.79, 1.07) (p<.001). Qualitative data supporting the quantitative findings will be presented. This study suggests that a video intervention may be an effective tool to increase knowledge and interest in palliative and end-of-life care among social work students.

